# *Notice to Readers*: *MMWR* Rural Health Series

**DOI:** 10.15585/mmwr.mm6602a7

**Published:** 2017-01-20

During 2017, *MMWR* will publish several Surveillance Summaries on topics that highlight rural health issues. The *MMWR* Rural Health Series is available at https://www.cdc.gov/mmwr/rural_health_series.html. Two recently published reports (*1*,*2*) described the death rate and number of potentially excess deaths from the five leading causes of death in the United States.
